# A nanoscale, multi-parametric flow cytometry-based platform to study mitochondrial heterogeneity and mitochondrial DNA dynamics

**DOI:** 10.1038/s42003-019-0513-4

**Published:** 2019-07-11

**Authors:** Julie A. MacDonald, Alisha M. Bothun, Sofia N. Annis, Hannah Sheehan, Somak Ray, Yuanwei Gao, Alexander R. Ivanov, Konstantin Khrapko, Jonathan L. Tilly, Dori C. Woods

**Affiliations:** 1 Department of Biology, Laboratory of Aging and Infertility Research, Northeastern University, Boston, MA 02115 USA; 2 Department of Chemistry and Chemical Biology, Northeastern University, Boston, MA 02115 USA; 3 Barnett Institute for Chemical and Biological Analysis, Northeastern University, Boston, MA 02115 USA

**Keywords:** Biological techniques, Cell biology, Mitochondria

## Abstract

Mitochondria are well-characterized regarding their function in both energy production and regulation of cell death; however, the heterogeneity that exists within mitochondrial populations is poorly understood. Typically analyzed as pooled samples comprised of millions of individual mitochondria, there is little information regarding potentially different functionality across subpopulations of mitochondria. Herein we present a new methodology to analyze mitochondria as individual components of a complex and heterogeneous network, using a nanoscale and multi–parametric flow cytometry-based platform. We validate the platform using multiple downstream assays, including electron microscopy, ATP generation, quantitative mass-spectrometry proteomic profiling, and mtDNA analysis at the level of single organelles. These strategies allow robust analysis and isolation of mitochondrial subpopulations to more broadly elucidate the underlying complexities of mitochondria as these organelles function collectively within a cell.

## Abstract (web summary)

Julie MacDonald et al. present a method for mitochondrial isolation using fluorescence-activated mitochondria sorting to facilitate analysis from individual organelles. They validate their platform using a number of downstream assays, including electron microscopy, proteomic profiling, and mtDNA analysis.

## Introduction

Aside from bioenergetics^1,2^, mitochondria coordinate ion flux, hormone synthesis, and apoptosis, all of which are central to aging^3^, cancer^4,5^, and cell fate determination^6^. Mitochondria function as coordinated heterogeneous populations and are not identical, even within a single cell. Variations in size, ultrastructure, mitochondrial membrane potential (Δψ_m_), and mitochondrial DNA (mtDNA) copy number exist, the regulation and purpose of which are poorly understood^7–10^. The absence of a method to identify and purify mitochondrial subtypes is a barrier to studying mitochondrial heterogeneity. Classical isolation techniques include gradient^11^ or differential^12^ centrifugation, and while such methods of isolation are successful at extracting bulk populations of mitochondria—these approaches mask any endogenous heterogeneity as a single sample. In addition, these approaches include both technical and practical pitfalls, including contamination of nonspecifically isolated organelles within the extracted sample^13^ and inconsistencies in manual sample preparations leading to problems with reproducibility^14^. In response to these well-reported limitations of classical isolation techniques, new approaches have been developed utilizing magnetic bead separations with antibodies against common mitochondrial proteins, such as TOM22^15,16^. However, while centrifugation-based techniques may be too broad and homogenizing, isolation of mitochondria based on a single antibody target is in all likelihood overly reductive.

Here we describe a novel technology for mitochondrial isolation using fluorescence-activated mitochondria sorting (FAMS). Mitochondria from diverse tissues and cell lines can be analyzed, isolated, and characterized based on multiple parameters, such as size, Δψ_m_, or protein markers, with as much diversity in experimental paradigm available as those seen for cell-level cytometry analyses. In addition, mitochondria purified by FAMS are energetically functional and a rich input source for proteomics profiling. We further demonstrate the utility of FAMS for single mitochondrion isolation, enabling studies of mtDNA sequences on a per mitochondrion basis. Although mitochondria function as a coordinated network^17^, the component members of a mitochondrial signaling network are not well understood, particularly with regard to the potential for distinct functional roles of mitochondrial subpopulations. The high level of resolution achieved with FAMS allows a more detailed understanding of mitochondria as individual organelles, as well as how mitochondrial subtypes relate to the more global function of mitochondria as a heterogeneous population.

## Results

### Nanoscale cytometry setup and technical validation

**Fig. 1:**
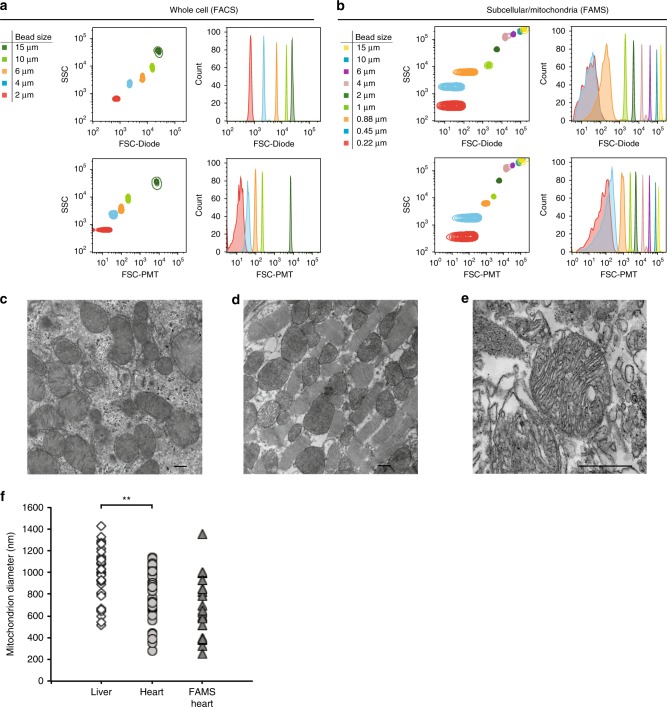
Size calibration of nanoparticle beads to optimize cytometer range for isolation of mitochondria from mouse tissues. **a** A custom FACS Aria III using a standard FSC-diode detector and voltages optimized for whole cell sorting can distinguish size particles down to 2 μm. **b** With the use of a FSC–PMT detector and voltages optimized for subcellular particles, the same instrument distinguishes nanoparticles from instrument noise down to 0.22 μm. **c**–**e** C57BL/6 mouse liver tissue (**c**), heart tissue (**d**), or mitochondria isolated from heart tissue by FAMS (**e**) were processed for evaluation of mitochondrial size by thin-section transmission electron microscopy (TEM). Representative images; scale bars, 500 nm. **f** Mitochondria identified by morphological evaluation of cristae structures were measured from random fields of view to determine appropriate size gates for analysis via FAMS and for size analysis of sorted events (liver tissue versus heart tissue: **P < 0.01; heart tissue versus FAMS-isolated heart mitochondria, no significant differences detected)

**Fig. 2:**
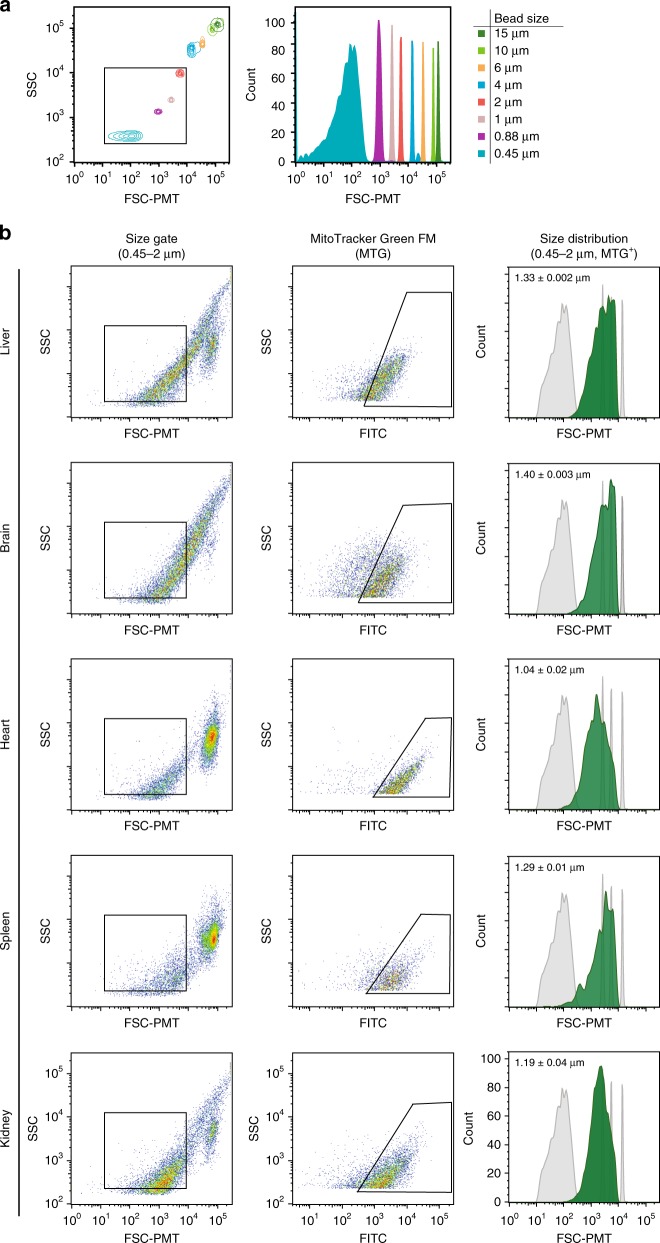
Mitochondrial isolation from mouse tissues using FAMS based on size and a mitochondrial-specific fluorescent probe. **a** Size calibration beads were used to distinguish events down to 0.45 μm. **b** In dissociated tissues stained with MitoTracker™ Green (MTG), events restricted via size gating to ~0.45–2 μm were assessed, and MTG-positive (MTG-labeled) events were detected in all samples analyzed (liver, brain, heart, spleen, kidney). MTG-positive events, shown in green, were assessed by FSC–PMT in reference to size calibration particles (shown in gray) to demonstrate size variability by tissue type, with the approximate mean diameter for each indicated; mean ± SEM, *n* = 3

To enable flow cytometric analysis of events at the size scale of mitochondria, we incorporated several design changes to a BD FACSAria III flow cytometer, including a ‘hybrid’ dual-forward scatter (FSC) mechanism consisting of a photomultiplier tube (PMT) and a diode, to distinguish small particles from debris and background instrument noise (Fig. 1). Initial studies with calibrated microbeads showed that under standard conditions for analyzing whole cells, resolution of the FSC parameter is limited to ~2.0 μm (Fig. 1a); however, following calibration of the FSC–PMT, we could reliably segregate individual events down to 0.22 μm (Fig. 1b). Mitochondria within liver (Fig. 1c) and heart (Fig. 1d) tissue of C57BL/6 mice, as well as mitochondria isolated from heart via FAMS (Fig. 1e), were processed for evaluation of mitochondrial size by thin-section transmission electron microscopy (TEM) to determine an appropriate size range for subcellular gating using FAMS. Liver mitochondria were significantly larger than those observed in heart (Fig. 1f: liver: 962.49 ± 39.78 nm; heart: 787.35 ± 37.56 nm; *P* < 0.01). By comparison, mitochondria isolated from heart by FAMS (Fig. 1e; 678.87 ± 61.11 nm) were not statistically different in size when compared to size estimates made from analysis of intact heart tissue. These data allowed us to establish a size-gating ‘standard curve’ for flow cytometry that fully covered the anticipated size range of mitochondria (0.45–2.0 μm) within the total cellular debris following lysis (Fig. 2a).

### Mitochondria assessment via size and fluorescent dyes

When our strategy was applied to cellular homogenates labeled with MitoTracker^TM^ Green (MTG)—a fluorescent probe that labels mitochondria irrespective of membrane polarization (Δψ_m_) status and is retained in mitochondria after cell lysis (Supplementary Fig. 1), we could reliably identify, analyze, quantitate, and sort mitochondria into purified fractions (Fig. 2). Plots shown highlight MTG-labeled (FITC-positive) events within the established size gate (Fig. 2a) and are representative of samples prepared from adult mouse liver, brain, heart, spleen, and kidney (Fig. 2b). The plots also show the size-range distribution across the total population of MTG-positive events in each tissue sample, based on FSC–PMT light scatter properties with the average estimated particle size between 1.04 ± 0.02 μm and 1.40 ± 0.003 μm (Fig. 2b).

### Validation of FAMS isolated events by mitochondrial properties

**Fig. 3:**
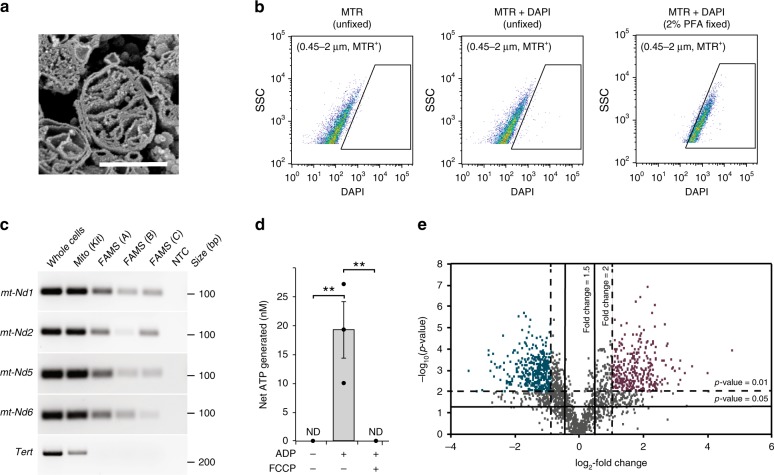
Analysis and characterization of FAMS-isolated mitochondria. **a** Mitochondria sorted from liver tissue exhibited intact outer and inner membranes, as well as intact cristae structures, when evaluated by scanning electron microscopy (SEM). Scalebar, 500 nm. **b** mtDNA was detected by DAPI-positive fluorescence in MTR-positive liver tissue lysates stained following fixation. **c** Size-gated, MTG-positive events (FAMS replicates A–C) expressed the mtDNA encoded genes, *mt-Nd1*, *mt-Nd2*, *mt-Nd5*, and *mt-Nd6*, but not *Tert*, a nuclear-encoded gene. Mitochondria isolated using a commercially available isolation kit (differential centrifugation) exhibit variable mtDNA purity. NTC, ‘no template’ control. **d** ATP generation by MTG-positive events after addition of ADP without and with carbonyl cyanide 4-(trifluoromethoxy) phenylhydrazone (FCCP) treatment. ND none detected; mean ± SEM, *n* = 3 (**P *<* 0.01). **e** Volcano plot of nLC-MS/MS-identified proteins from size-gated MTG-positive events collected from liver and brain tissues; colors indicate enrichment in either liver (purple) or brain (blue)

To verify that FAMS was accurately identifying and successfully isolating mitochondria, mouse liver homogenates were prepared and MTG-labeled events in the 0.45–2.0 μm size gate were collected for several downstream analyses (Fig. 3). Initially, the post sort samples were processed for scanning electron microscopy (SEM)^18^ by freeze fracture to visualize internal mitochondrial ultrastructural properties^10^. This confirmed the MTG-positive events were mitochondria that possessed structurally intact outer and inner membranes, as well as intact internal cristae (Fig. 3a). We then used MitoTracker™ Red CMXRos (MTR)—a Δψ_m_-sensitive probe that is retained following aldehyde fixation, in combination with 4′,6-diamidino-2-phenylindole dihydrochloride (DAPI) to confirm the presence of DNA within events identified as mitochondria by FAMS. Analysis of MTR-stained hepatocytes that were lysed, fixed, and labeled with DAPI demonstrated the total MTR-positive population shifted to include DAPI-positive fluorescence over unfixed controls (Fig. 3b). Furthermore, mitochondrial fractions isolated by FAMS contained mtDNA sequences (*NADH dehydrogenases 1, 2, 5* and *6*; *mt-Nd1*, *mt-Nd2*, *mt-Nd5*, and *mt-Nd6)* but not nuclear-encoded genomic sequences (*i.e*., *telomerase reverse transcriptase* or *Tert)* (Fig. 3c), thus tying the DAPI fluorescence detected in MTR-positive events to mtDNA (Fig. 3c and Supplementary Fig. 2). Of note, these studies also demonstrated contaminating nuclear-encoded DNA in the mitochondria isolated using a commercially available differential centrifugation-based isolation kit (Fig. 3c).

To test for functional competence, mitochondria isolated from mouse liver by FAMS were analyzed using a luminescence-based ATP-generation assay. As a negative control, isolated mitochondria did not generate detectable levels of ATP in the absence of ADP substrate. However, inclusion of ADP in the mitochondrial fractions resulted in 19.27 ± 4.87 nM of ATP production (Fig. 3d), which was reduced to nondetectable levels when samples were preincubated with the electron transport chain uncoupler, carbonyl cyanide 4-(trifluoromethoxy) phenylhydrazone (FCCP), prior to the addition of ADP (Fig. 3d). Similar results were obtained using mitochondria isolated from mouse embryonic fibroblasts (MEFs), in which only those samples provided with ADP generated ATP in an FCCP-sensitive manner (Supplementary Fig. 1).

As a final validation strategy, mitochondria isolated from mouse liver, or brain were processed for tandem mass spectrometric analysis of quantitative proteomic profiles through nLC-MS/MS (Fig. 3e). A total of 1853 proteins were identified across the liver and brain mitochondrial samples (Supplementary Data). An overrepresentation test of the identified proteins revealed a clear enrichment of mitochondria-mediated biological processes, such as the mitochondrial carrier system (SLC25 family genes)^19^, as identified by the C4-dicarboxylate transport GO term, as well as effectors of oxidative phosphorylation (*Sdhb*, *Idh3g*, and *Mdh2*), as identified by the Tricarboxylinc acid cycle GO term^20^ (Table 1). A core proteome of 361 nondifferentially expressed proteins were present in mitochondria from both tissues, based on relative quantitation utilizing tandem mass tag (TMT)^21^ reporter ion intensities (Supplementary Data). In addition, we identified 243 proteins with elevated expression in liver versus brain mitochondria, and 1249 proteins with elevated expression in brain versus liver mitochondria (Fig. 3e), emphasizing the divergence of mitochondrial protein expression between tissues. Proteins with elevated expression in liver mitochondria were enriched for biological processes inclusive of the cytochrome P450 superfamily proteins expected to be enriched in liver mitochondria^22–24^, such as secondary metabolic process [*Cyp2a5, Cyp2a4, Cyp2a12*] and response to xenobiotic stimulus [*Cyp2e1, Cyp2c50, Cyp2c23, Cyp2f2, Cyp2c70, Cyp2a5, Cyp2c37, Cyp2c29, Cyp2a4, Cyp2a12, Cyp2d10, Cyp2c69*] (Table 2). In addition, proteins with elevated expression in brain mitochondria (Table 3) were enriched for processes such as protein targeting to mitochondrion, the tricarboxylic acid cycle, and mitochondrial fission, the latter of which includes ganglioside induced differentiation associated protein 1 (*Gdap1*), an essential regulator of mitochondrial fission^25,26^.

**Table 1:** Top 10 biological processes over-represented in FAMS-isolated mitochondrial proteomics

Panther go-slim biological process	Gene product
C4-dicarboxylate transport	Slc25a13, Slc25a12, Slc25a22, Slc25a18
Tricarboxylic acid cycle	Idh3g, Aco2, Sdhd, Suclg2, Idh3b, Mdh2, Suclg1, Idh3a, Cs, Ldhb, Mdh1
Inner mitochondrial membrane organization	Afg3l2, Timm13, Tmem11, Mic13, Timm10, Apool, Chchd6, Dnajc11
Mitochondrial electron transport, ubiquinol to cytochrome c	Uqcrq, Pmpcb, Uqcr10, Uqcrh
Protein targeting to mitochondrion	Timm13, Gdap1l1, Gdap1, Timm10
Mitochondrial transmembrane transport	Timm13, Mpc1, Slc25a13, Slc25a1, Slc25a12, Slc25a22, Slc25a18, Slc25a15, Timm10, Mcu, Mpc2
Mitochondrial fission	Gdap1l1, Dnm1l, Mtfr1l, Gdap1, Fis1
Fatty acid catabolic process	Hibch, Acat2, Eci1, Ehhadh, Acaa2, Acaa1a, Hacl1, Hadha, Echdc2, Etfa, Hadhb, Cpt2, Acox1
ATP synthesis coupled proton transport	Uqcrq, Cox7c, Pmpcb, Uqcr10, Atp5f1a, Sdhd, Ndufa10, Ndufs6, Uqcrh, Atp5l, Atp5mc2, Cox7a2, Cox5a
Long-term synaptic potentiation	Grin2a, Shank1, Calb2, Calb1, Shank3

**Table 2:** Top 10 biological processes over-represented in proteins overexpressed in liver FAMS-isolated mitochondria relative to brain

Panther go-slim biological process	Gene product
Secondary metabolic process	Akr1c6, Cyp2a5, Cyp2a4, Cyp2a12
Response to xenobiotic stimulus	Cyp2e1, Cyp2c50, Cyp2c23, Ugt1a5, cyp2j5, Cyp2f2, Cyp2c70, Cyp2a5, Cyp2c37, Cyp2c29, Cyp2a4, Ugt1a9, Cyp2a12, Cyp2d10, Cyp2c69
Fatty acid catabolic process	Ehadh, Hacl1, Hadha, Etfa, Hadhb, Acox1
Protein targeting to ER	Sec61b, Sec61a1, Spcs3
Mitochondrial transmembrane transport	Mpc1, Slc25a13, Slc25a15, Mpc2
Response to drug	Cyp2e1, Cyp2c50, Cyp2c23, Cat, Cyp2j5, Cyp2f2, Cyp2c70, Cyp2a5, Cyp2c37, Cyp2c29, Cyp2a4, Cyp2a12, Cyp2d10, Cyp2c69
Cellular response to chemical stimulus	Prdx4, Cyp2e1, Cyp2c50, Cyp2c23, Ugt1a5, Cat, Cyp2j5, Cyp2f2, Cyp2c70, cyp2a5, Cyp2c37, Cyp2c29, Cyp2a4, Ugt1a9, Cyp2a12, Cup2d10, Cyp2c69,
Drug metabolic process	Prdx4, Gldc, Cyp2e1, Cyp2c50, Cyp17a1, Cyp2c23, Akr1c6, cat, Cyp2j5, Cyp2f2, Cyp2c70, Cyp2a5, Cyp2c37, Cyp2c29, Cyp2a4, Cup2a12, Cyp2d10, Cyp2c69
Cellular amino acid biosynthetic process	Otc, Cps1, Acsf2, Ass1
Respiratory electron transport chain	Cyp27a1, Slc25a13, Cyp3a41a, Maob, Cyp5f14, Chdh, Cyp4a14

**Table 3:** Top 10 biological processes overrepresented in proteins overexpressed in brain FAMS-isolated mitochondria relative to liver

Panther go-slim biological process	Gene product
C4-dicarboxylate transport	Slc25a12, Slc25a22, Slc25a18
Protein targeting to mitochondrion	Timm13, Gdap1l1, Gdap1, Timm10
Tricarboxylic acid cycle	Idh3g, Aco2, Idh3b, Mdh2, Suclg1, Idh3a, Cs, Ldhb, Mdh1
Vesicle transport along microtubule	Pafah1b1, Dync1h1, Ndel1
Long-term synaptic potentiation	Grin2a, Shank1, Calb2, Shank3
Myelination	Mal, Pllp, Mal2
Mitochondrial fission	Gdap1l1, Dnm1l, Gdap1, Fis1
Inner mitochondrial membrane organization	Afg3l2, Timm13, Mic13, Timm10, Chchd6
Septin ring organization	Sept6, Sept4, Anln, Sept3, Sept5
Amino acid transport	Slc6a17, Slc1a6, Slc1a1, Slc1a3, Slc1a2

### Protein expression of mitochondrial subpopulations

**Fig. 4:**
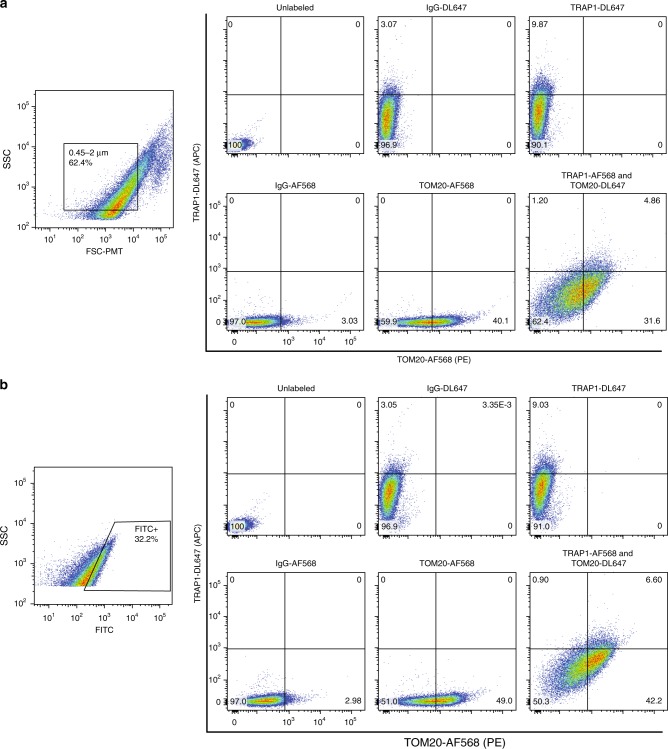
FAMS analysis of mitochondrial subpopulations in mouse liver tissue via TOM20 and TRAP1 antibody labeling. **a** Mouse liver cells were stained with MTG followed by labeling with anti-TOM20, anti-TRAP1, and AlexaFluor 568 or DyLight 647-conjugated secondary antibodies, respectively. Lysates were analyzed for TOM20 and TRAP1 colabeling of mitochondria using only a 0.45–2 μm size parental gate, or **b** through the 0.45–2 μm size gate in addition to a parental gate for MTG-positive events

To highlight potentially unexplored degrees of mitochondrial heterogeneity within a single tissue type at the level of protein expression, we evaluated expression of two proteins identified by proteomics using antibody-based labeling and FAMS analysis. A well-characterized outer mitochondrial membrane protein, TOM20 (ref.^27^) (Supplementary Data), was used to identify mitochondria and demonstrated the benefit of subjecting samples to both a size gate and a mitochondrial dye, like MTG, to reduce background cellular debris in the analyses (Fig. 4a, b). A lesser-characterized mitochondrial protein, TRAP1 (Supplementary Data), has been shown to primarily localize within the inner membrane and cristae of mitochondria^28^; however, some studies have reported expression of TRAP1 at the outer mitochondrial membrane^29^. Antibody-based labeling identified a small subset of TRAP1-positive events colabeled with TOM20, in support of both structurally intact mitochondrial samples as well as differential localization of TRAP1 within the total mitochondrial population (Fig. 4). This experiment exemplifies that a simple antibody labeling experiment identifies four possible subtypes of mitochondria, with the potential to isolate these individual populations to perform comparative functional analyses. Parameters used to identify the total number of mitochondrial subpopulations are limited only by the multiparameter detection capabilities of the instrument used.

### Differential Δψ_m_ of mitochondrial subpopulations

**Fig. 5:**
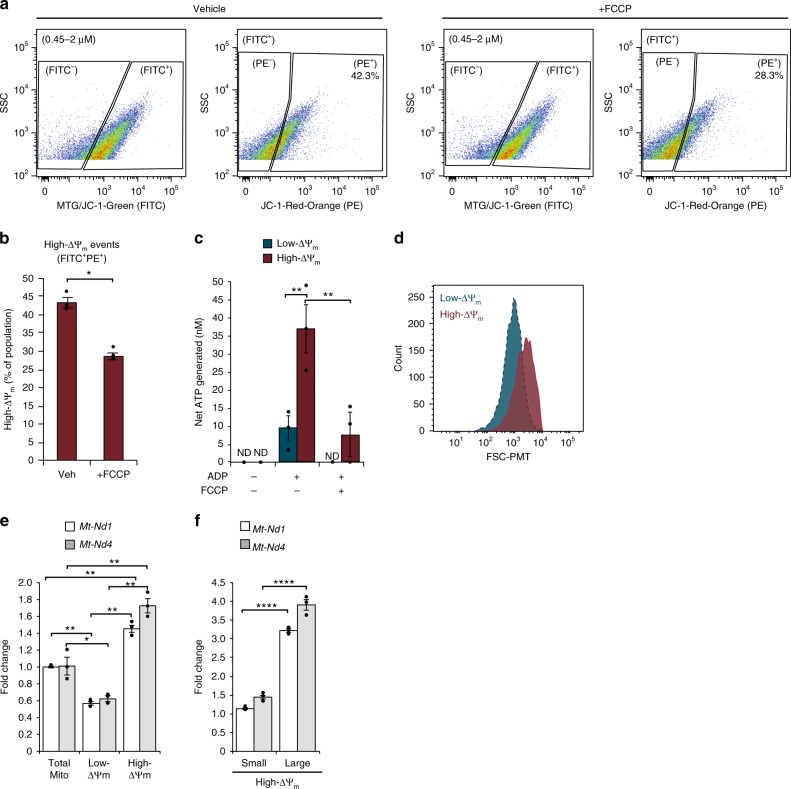
Characterization and analysis of mitochondrial subpopulations by ΔΨ_m_ isolated by FAMS. **a** PE^+^ events were gated from size and FITC^+^ [MTG/JC1-Green] events, indicative of high-ΔΨ_m_ mitochondria. **b** FCCP significantly reduced the number of high-ΔΨ_m_ mitochondria, mean ± SEM, *n* = 3 (*P* < 0.05*). **c** High-ΔΨ_m_ (FITC^+^PE^+^) mitochondria generated significantly more ATP than low-ΔΨ_m_ (FITC^+^PE^−^) mitochondria, and ATP production was significantly reduced after preincubation with FCCP. ND none detected; mean ± SEM, *n* = 3 (*P* < 0.01**). **d** High-ΔΨ_m_ and low-ΔΨ_m_ mitochondrial subpopulations were assessed for size distribution based on FSC–PMT (representative histogram for *n* = 3). **e** Each ΔΨ_m_ subpopulation was assessed for levels of *mt-Nd1* and *mt-Nd4* by qPCR, mean ± SEM, *n* = 3 (*P* < 0.05*, *P* < 0.01**). **f** High-ΔΨ_m_ mitochondria were further segregated into small and large populations and assessed for levels of *mt-Nd1* and *mt-Nd4* by qPCR, mean ± SEM, *n* = 3 (*P* *<* 0.0001****)

The cationic mitochondrial membrane potential sensor dye, JC-1, was then used to distinguish subpopulations of mitochondria (Fig. 5). The characteristic shift in fluorescence emission from ~525 nm (FITC) at low concentrations to ~590 nm (PE) following the formation of red-orange–fluorescing JC-1 aggregates is dependent on Δψ_m_, and thus can differentiate subpopulations within the total mitochondrial population. As the nonaggregate form does not accumulate within mitochondria, total mitochondria were assessed using MTG. Because the fluorescence of MTG and the nonaggregated JC-1 (~525 nm) are detected with the same filter set (FITC), we utilized a binary classification of either low-Δψ_m_ (FITC^+^PE^−^) or high-Δψ_m_ (FITC^+^PE^+^) events. Of the total mitochondrial population identified by size and labeling with MTG as described above, 43.2 ± 1.5% exhibited high-Δψ_m_ (Fig. 5a), which was reduced to 27.8 ± 1.0% following addition of FCCP (Fig. 5a, b; *n* = 3, *P* < 0.05). These results were then verified using tetramethylrhodamine methyl ester (TMRM), a different Δψ_m_-dependent dye, using both FCCP and valinomycin controls (Supplementary Fig. 3). In the glycolytic human liver carcinoma cell line, HepG2, 33.7 ± 4.4% of the total mitochondrial events were FITC^+^PE^+^ using JC-1, and this was reduced to 6.7 ± 2.2% following treatment with FCCP (Supplementary Fig. 1; *n* = 3, *P* < 0.05). Both low-Δψ_m_ and high-Δψ_m_ mitochondrial populations isolated from primary hepatocytes generated ATP when provided with ADP substrate (Fig. 5c); however, high-Δψ_m_ mitochondria produced more ATP (31.38 ± 5.78 nM) than low-Δψ_m_ mitochondria (5.97 ± 2.63 nM) (*n* = 3, *P* < 0.01). Utilizing FSC–PMT values as one-dimensional proxies for particle size ranges, we noted a significant difference in the size range between the low-Δψ_m_ and high-Δψ_m_ mitochondrial subpopulations (Fig. 5d). The mean FSC–PMT signal of low-Δψ_m_ mitochondria was 1020 ± 35 AU, whereas for high-Δψ_m_ mitochondria it was 2500 ± 46 AU (*n* = 3, *P* < 0.001). These data show that mitochondrial subpopulations with variable Δψ_m_ are present within a single biological sample. Furthermore, these mitochondrial subpopulations are functionally and morphologically distinct and, as such, can be isolated by FAMS to be studied as independent entities based on these properties.

**Fig. 6:**
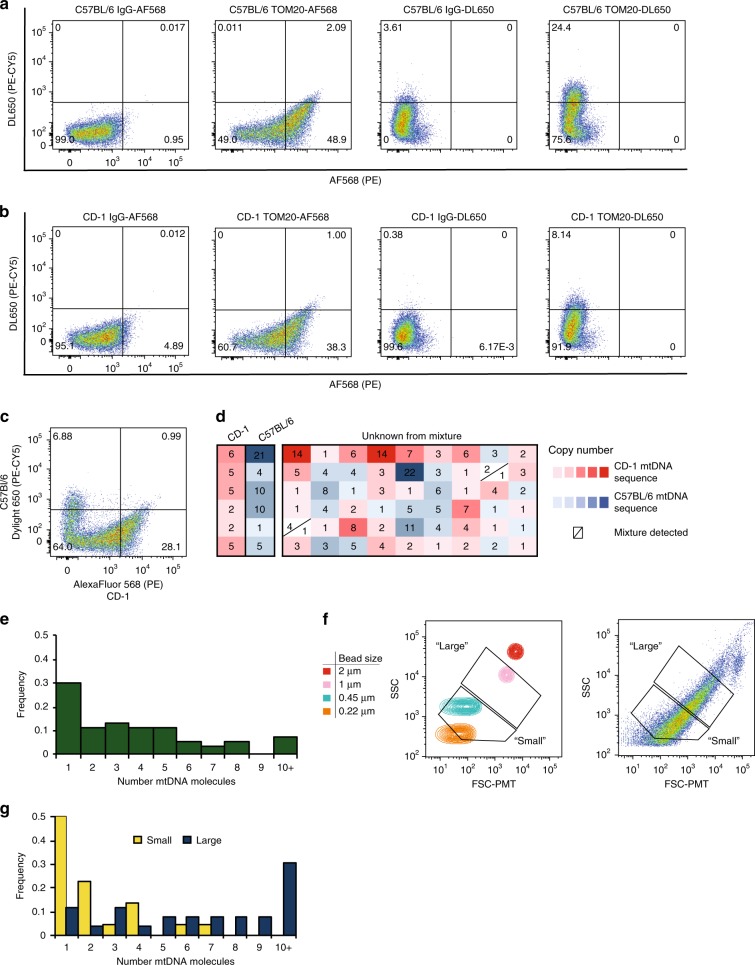
Analysis of mitochondrial heterogeneity by single organelle isolation and single molecule PCR. Mouse liver cells were stained with MTG and labeled with anti-TOM20 and AlexaFluor 568 or DyLight 650. Lysates were analyzed by size gating through 0.45–2 μm, followed by gating MTG-positive events. **a** C57BL/6 cell lysates were labeled with either secondary antibody, and gates were set based on isotype matched controls. **b** CD-1 lysates were labeled with either secondary antibody and gates were set based on isotype matched controls. **c** Following MTG staining, anti-TOM20 immunolabeling, and strain-specific secondary antibody labeling (CD-1:AF568, C57BL/6:DL650) separable populations could be resolved from mixed-strain samples. **d** Individual mitochondria were analyzed for mtDNA copy number. Each box represents a single mitochondrion with strain identified by sequencing (red:CD-1; blue:C57BL/6; white indicates mixture) and color-intensity associated with mtDNA copy number (light, low copy number; dark, high copy number). **e** Single mitochondria sorted from mixed strain samples based only on MTG-staining contained 1–10+ mtDNA molecules. **f** To sort single mitochondria from specific size ranges, size gates were determined based on nanoparticles to select events corresponding to ~0.22–0.5 μm (small) or 0.5–1 μm (large) mitochondria. **g** Mitochondrial copy number was calculated for individual mitochondria sorted from small or large size gates

To further characterize subpopulations based on low versus high Δψ_m_, mitochondria were collected for mtDNA copy number analysis by both quantitative PCR (qPCR; using primers targeting *mt-Nd1* and *mt-Nd4*) (Fig. 5e) and single molecule PCR (smPCR; Fig. 6). When compared with mtDNA copy number in the total pool of mitochondria (not sorted based on Δψ_m_), we found that levels of *mt-Nd1* and *mt-Nd4* were reduced by 43 ± 4% and 39 ± 6%, respectively, in the low-Δψ_m_ subpopulation (*n* = 3, *P* < 0.05 versus total pool). In turn, levels of *mt-Nd1* and *mt-Nd4* were elevated 1.45 ± 0.04-fold and 1.72 ± 0.08-fold, respectively, in the high-Δψ_m_ subpopulation(*n* *=* 3, *P* < 0.01 versus total pool) (Fig. 5e). These differences were even more pronounced when an additional size parameter was imposed on the high-Δψ_m_ subpopulation to separately collect small:high-Δψ_m_ and large:high-Δψ_m_ mitochondria. Compared to the total mitochondrial pool, the small:high-Δψ_m_ mitochondria showed a modest increase in *mt-Nd1 and mt-Nd4* levels (1.14 ± 0.03-fold for *mt-Nd1*, *n* = 3, *P* < 0.01; 1.45 ± 0.06-fold for *mt-Nd4*, *n* = 3, *P* < 0.01) (Fig. 5f); however, the large:high-Δψ_m_ subpopulation showed higher levels of both *mt-ND1* and *mt-Nd4* compared with the total pool (3.21 ± 0.04-fold for *mt-Nd1*, *n* = 3, *P* < 0.001; 3.90 ± 0.15-fold for *mt-Nd4*, *n* = 3, *P* < 0.001) (Fig. 5f). Interestingly, the elevated mtDNA copy number in small:high-Δψ_m_ as compared to low-Δψ_m_ of approximately the same small size range indicates that Δψ_m_, in addition to mitochondrial size, may be an important parameter for studying mtDNA dynamics.

### Heterogeneity analysis via single organelle isolation

In a final set of experiments, we tested the utility of FAMS to study mtDNA copy number, which is a widely reported feature of mitochondrial heterogeneity^30–32^, on a single mitochondrion level (Fig. 6). To ensure that mitochondria were reliably sorted and collected as individual organelles, we first validated our strategy using samples prepared from C57BL/6 and CD-1 strains of mice, such that a C57BL/6-specific mtDNA polymorphism (C9461T) could be utilized to identify strain-specific mtDNA following sequence analysis. Liver samples from each strain were labeled with MTG, and independently tagged with TOM20 antibody followed by either AF568 (CD-1; PE channel) or DL650 (C57BL/6; PE-CY5 channel) secondary antibody. Sorts were then conducted on individual strain samples to generate positive controls for strain identification by smPCR (Fig. 6a, b) or on mixed samples (1:1 ratio of each strain) to determine the accuracy of simultaneously sorting single mitochondria from multiple populations within one sample (Fig. 6c). Of the positive control molecules sequenced for each strain (C57BL/6: *n* = 21; CD-1: *n* = 7), the identified mtDNA sequence matched the sorted fluorescent label with 100% fidelity (Fig. 6d). The lack of a single cross-strain contaminant indicates that FAMS can be used to sort and collect individual mitochondria, with no artificial fusion of mitochondria following cell lysis during sample acquisition or downstream smPCR.

We then analyzed mtDNA copy number in single mitochondria, and included mitochondrial size as a parameter to distinguish subpopulations. Single mitochondria were isolated from samples prepared from a mixture of C57BL/6 and CD-1 liver tissue labeled only with MTG. A total of 59 sorted mitochondria from the strain mixture were collected individually, and mtDNA was amplified from each mitochondrion by smPCR. From these mitochondria, 246 mtDNA molecules were obtained and sequenced, with copy numbers ranging from 1 to 22 molecules per sorted event (Fig. 6e). The total mitochondrial population was non-normally distributed (D’Agostino–Pearson omnibus K2 test for normality, *P* < 0.0001) and contained a median number of three copies of mtDNA per mitochondrion (Fig. 6e). To isolate mitochondria by size, we used calibrated microparticles to further refine two size gates corresponding to ~0.22–0.5 μm (small) or 0.5–1 μm (large) (Fig. 6f). Doing this, we found that mtDNA copy number in small size-gated mitochondria was significantly lower in both CD-1 (median copy number of 2) and C57BL/6 (median copy number of 1) when compared to the large size-gated mitochondria from each strain (median copy number for CD1 of 7.5, *P* < 0.01; median copy number for C57BL/6 of 6.5, *P* < 0.001). In addition, mtDNA copy number in size-populations did not differ between mouse strains (small size: *P* = 0.1216; large size: *P* = 0.4521). While traditional qPCR studies of mtDNA copy number using pooled mitochondrial samples (see Fig. 5e, f for example) allow estimations of mtDNA properties, single organelle isolation via FAMS allows for direct evaluation of mtDNA at the level of individual mtDNA molecules within a mitochondrion.

## Discussion

Collectively, this work has established a powerful and broadly adaptable nanocytometry-based strategy to isolate mitochondria, as well as mitochondrial subpopulations, based on several parameters previously associated with mitochondrial heterogeneity in cells and tissues. Following isolation, sorted mitochondria were structurally intact, contained mtDNA detectable by both DAPI labeling as well as postisolation PCR, and were competent to generate quantifiable levels of ATP. Comparative proteomic analysis of mitochondria isolated from both brain and liver tissue identified potential protein-based targets for analysis of subpopulations, and as proof of principle, differential expression of TRAP1 was demonstrated via immunolabeling. In addition, functionally distinct subpopulations based on differences in Δψ_m_ were validated by ATP generation assays, and were significantly different in both size as well as mtDNA content. To further investigate inherent differences of individual mitochondria, single organelles were collected and analyzed by smPCR. This approach revealed that significant differences in mtDNA content were correlated with mitochondrial size, and this was consistent between two mouse strains. This ability to study individual organelles will enable future investigations of mtDNA heteroplasmy and dynamics that have historically proven impossible to pursue. Moreover, since FAMS can be readily applied to the study of multiple cell and tissue types, even those of limited availability, this technology will enable unprecedented exploration into the drivers of differences across mitochondrial subpopulations in both form and function, and how these differences contribute to changes in cell function during fate determination, differentiation, aging, and disease.

## Methods

### Animals and cell lines

All animals utilized for this study were young adult (8–12-week-old) female C57BL/6 or male CD-1 mice (Charles River Laboratories), and all experiments described herein were reviewed and approved by the Institutional Animal Care and Use Committee of Northeastern University. Mouse tissues were harvested and immediately dissociated into single-cell suspensions for mitochondrial preparations. The human liver hepatocellular carcinoma cell line, HepG2 was obtained from ATCC (ATCC^®^ HB-8065™) and maintained as recommended by the supplier. CF-1 MEFs were obtained from MTI-GlobalStem (GSC-6001) and maintained as recommended by the supplier. Neither cell line is included in the ICLAC register for commonly misidentified lines nor authenticated or tested for mycoplasma contamination. All reagents were purchased from Thermo Fisher Scientific unless otherwise noted.

### Tissue dissociation into single cell suspension

Harvested tissues (300–1200 mg) were rinsed briefly in warm Hank’s balanced salt solution with calcium and magnesium (HBSS^+/+^) and minced into small pieces. For MitoTracker^*™*^ Green FM (MTG; Life Technologies) labeling experiments, minced tissue (liver, heart, spleen, brain, and kidney) was placed in PBS containing 2 mM EDTA and 0.5% bovine serum albumin (BSA) (PEB Buffer) and mechanically dissociated using a gentleMACS Tissue Dissociator (Miltenyi). For further subpopulation analyses and sample collection for proteomics analyses, liver or brain tissue was minced and placed in a tissue-specific dissociation buffer. Liver dissociation buffer contained 500 U ml^−1^ collagenase IV (Worthington Biochemical Corporation) and 150 U ml^−1^ DNaseI (Sigma Aldrich) in HBSS^+/+^, and brain dissociation buffer contained PBS with 2 mM EDTA. Tissues were incubated at 37 °C with continuous agitation for 30 min and then mechanically dissociated using a gentleMACS Tissue Dissociator. All samples were filtered through a 100-μm nylon-mesh cell strainer, rinsed with PEB buffer, and maintained at 4 °C prior to labeling.

### MitoTracker^™^, DAPI, JC-1, and TMRM labeling

Cultured cells were incubated with trypsin and brought to a single cell suspension in PBS containing 1% BSA and 2.5 mM EDTA (FACS Buffer). Whole cells were stained with 100 nM MTG, 2 μM JC-1 (Marker Gene Technologies), or both dyes simultaneously at 37 °C for 15 min. Dispersed liver or brain tissue was stained with 100 nM MTG for sorting whole populations of mitochondria. After mitochondrial labeling, cell suspension samples were briefly pelleted and resuspended in ice-cold cell lysis buffer containing 300 mM sucrose, 10 mM Tris (pH 7.4), 0.5 mM EDTA, and 1× Halt Protease Inhibitor Cocktail in PBS. Tissue samples were lysed by vortexing, while HepG2 cells and MEFs were dounce homogenized on ice for lysis.

In other studies, a single cell suspension from mouse liver tissue was stained with 25 nM MitoTracker^™^ Red CMXRos (MTR; Life Technologies) and lysed as described above. Lysed cell samples were fixed in 2% paraformaldehyde for 5 min at room temperature, rinsed with PBS, and subsequently stained with 3 μM DAPI for 5 min at room temperature. After a final rinse, samples were analyzed for staining of mtDNA within the population of size-gated MTR^+^ events.

For mitochondrial subpopulation sorting from liver tissue based on JC-1, dispersed cell suspensions were simultaneously stained with 25 nM MTG and 1 μM JC-1 for 15 min at 37 °C (under 5% CO_2_). To assess the specificity of JC-1 labeling, stained cells were incubated with 10 μM carbonyl cyanide 4-(trifluoromethoxy)phenylhydrazone (FCCP; Tocris) for 15 min at 37 °C (under 5% CO_2_) prior to lysis.

For mitochondrial subpopulation sorting from liver tissue based on TMRM, dispersed cell suspensions were swelled using a hypotonic buffer^33^ followed by lysis with a dounce homogenizer. Lysed samples were pelleted via centrifugation at 12,000 × *g* for 5 min. Pellets were resuspended in mitochondrial respiration buffer^34^ (225 mM d-mannitol, 75 mM sucrose, 10 mM KCl, 10 mM Tris-HCl, 5 mM KH_2_PO_4_, pH 7.2) and stained with 25 nM MTG and 100 nM TMRM for 15 min at room temperature. To assess the specificity of TMRM labeling, stained samples were incubated with 50 μM FCCP or 50 μM valinomycin (Tocris) for 1 h at room temperature prior to analysis against vehicle-treated controls.

### Antibody labeling

A single cell suspension from mouse liver tissue was stained with 25 nM MTG as described above and lysed in cell lysis buffer. Samples were centrifuged at 12,000 × *g* for 5 min at 4 °C, resuspended in blocking buffer (2% BSA and 2% normal goat serum in PBS), and incubated on crushed ice for 20 min. Samples were centrifuged at 12,000 × *g* and then reacted with anti-TOM20 antibody (Santa Cruz Biotechnology; sc-11415, clone FL-145, Lot#L1713), anti-TRAP1 (Abcam; ab2721, Lot#GR219935-6), both antibodies together, or matched isotype controls (mouse IgG, Invitrogen; rabbit IgG, Invitrogen). Primary antibodies against TOM20 and TRAP1 were validated by the manufacturers using positive control western blots (by size) in both human and mouse samples. Samples were washed in ice-cold PBS and resuspended with goat anti-mouse AlexaFluor 647 (Molecular Probes, 4410S, Lot#10, 1:100), goat anti-rabbit AlexaFluor 568 (Life Technologies, A11011, Lot#1942295, 1:100), or both secondary antibodies together, and incubated on ice for 20 min. Wash steps were repeated and samples were resuspended in cold (4 °C)  saline sheath fluid (blood bank saline) for FAMS analysis. In a separate experiment to identify mitochondria from a mixed pool of mouse strains, mouse liver homogenates from CD-1 and C57BL/6 strains were incubated with anti-TOM20 antibody, followed by incubation with either AF568 (for CD-1) or DL650 (for C57BL/6) secondary antibodies. The samples were washed, combined at a 1:1 ratio and resuspended in cold sheath fluid for FAMS analysis.

### Fluorescence-activated mitochondria sorting (FAMS)

All analyses were completed using a special order research product BD FACS Aria III, fitted with a PMT detector for forward light scatter (FSC) of a 488 nm laser, allowing for a higher dynamic range of small particle detection over standard photodiode detectors. To achieve high-resolution detection of subcellular-sized calibration beads (<1 μm), the instrument detection threshold was routinely set to side light scatter (SSC) 200. The use of fluorescently labeled size calibration particles (Spherotech), which were initially identified and gated as FITC-positive, allowed for accurate identification of subcellular-sized particles in what would otherwise be considered the debris field, or instrument noise, under standard cell sorting parameters (Fig. 1a, b). Attempts to precisely measure the size of a complex biological particle based on the use of solid polystyrene calibration particles should be interpreted with some degree of caution, however, since the refractive index of engineered microparticles can be orders of magnitude different from that of intact mammalian cells^35^; this has direct implications for light scattering properties. However, previous studies have shown that mitochondria have refractive indices more similar to those of sizing particles when compared to whole cells^35^. Due to these limitations, we utilized calibration beads to define an appropriate size range for sorting mitochondria, as well as to estimate mitochondrial size from FSC–PMT values using a linear fit of known microparticle size and mean fluorescence intensity of FSC–PMT. In addition, for any gating parameters used in this study to isolate mitochondrial subpopulations by size, two-dimensional gates were used incorporating both FSC–PMT and the more sensitive SSC.

Following standard instrument calibration of laser area scaling and delay, for each sort a mixture of size calibration beads (Spherotech and Life Technologies) was run to optimize light scatter voltages for visualization of events between 0.22 and 6 μm. Typical size gates for sorts of mouse liver mitochondria capturing particles ranging in size from 0.45 to 2 μm were based on TEM evaluation of mitochondrial size in situ (Fig. 1).

Mitochondria were identified by size and by positive fluorescence using mitochondrial-specific dyes, such as MTG, or by secondary antibody fluorophore (with respect to unstained negative control samples or control samples labeled using isotype controls for the respective primary antibody), allowing up to 5% false positive events versus the negative control sample. For the MTG gate, an unstained sample was used to establish a negative population with respect to MTG(FITC)-positive events. A gate was then drawn to exclude all but up to 0.5% false positive events, to correct for experimental variation. In experiments utilizing multicolor detection, spectral overlap was corrected, as needed, via manual compensation using single color samples. Compensation was not applied to samples labeled with JC-1, due to emission properties of the dye. For all analyses, at least 3 × 10^4^ events were collected per sample; data were acquired using BD FACSDiva software (version 8.0.1) and then analyzed using Microsoft Excel (version 15.27) or Graphpad Prism (Version 7.0d) and FlowJo (version 10). We observed a sort rate of ~15 min to collect 4.5 × 10^6^ events and during this time samples were maintained in sucrose buffer diluted 1:5 with saline sheath fluid [60 mM sucrose, 2 mM Tris (pH 7.4), 0.1 mM EDTA, 0.2× Halt Protease Inhibitor Cocktail], followed by centrifugation to concentrate samples when required. Future studies may benefit from a more appropriate cytometer sheath +fluid to better maintain mitochondrial morphologies; however, EM analysis and functional testing (Figs. 3d and 5c) supports maintenance of intact mitochondria throughout process of FAMS.

### Transmission electron microscopy (TEM)

Tissue harvested from 12-week-old C57BL/6 mice was dissected into 1 mm^3^ pieces. After dissection, samples were fixed with 2.5% glutaraldehyde and 2.5% formaldehyde (Electron Microscopy Sciences) in 0.1 M sodium cacodylate buffer (pH 7.2) (Electron Microscopy Sciences) overnight at 4 °C. After fixation, the samples were rinsed in 0.1 M sodium cacodylate buffer and postfixed in 1.0% osmium tetroxide (Electron Microscopy Sciences) in 0.1 M sodium cacodylate buffer for 2 h at room temperature. Following a second rinse, the samples were dehydrated through a graded ethanol series. Once dehydrated, the samples were infiltrated with Spurr’s low-viscosity resin (Electron Microscopy Sciences) supplemented with quetol (Electron Microscopy Sciences) and embedded. Ultrathin sections were cut on a LKB Ultrotome III and mounted onto 200-hex mesh copper grids (Electron Microscopy Sciences). The sections were exposed to a primary stain of 5% aqueous uranyl acetate (Electron Microscopy Sciences) and, after rinsing, underwent a secondary stain using Reynolds’ lead citrate (Electron Microscopy Sciences). Samples were then analyzed using a JEOL JEM1010 transmission electron microscope. For quantitation of mitochondrial size, 5–10 random fields of view were imaged for each sample (liver, heart, FAMS-isolated heart mitochondria). Mitochondria were identified based on morphology and sizes of individual mitochondria in each sample (liver tissue: *n* = 36; heart tissue, *n* = 36; FAMS-isolated heart mitochondria, *n* = 20) were measured using the average of cross-sectional diameters with ImageJ (version 1.49).

### Scanning electron microscopy (SEM)

Size-gated MTG-positive events (3 × 10^7^) were pelleted and placed in a primary fixative composed of 2.5% glutaraldehyde and 2.5% formaldehyde in 0.1 M sodium cacodylate buffer (pH 7.2) followed by postfixation in 1.0% osmium tetroxide in sodium cacodylate buffer. The samples were then freeze fractured in liquid nitrogen as described previously^10,18^, processed through a second postfixation cycle, and impregnated with osmium tetroxide after a period of incubation with 1.0% tannic acid in sodium cacodylate buffer. Some fractured samples were embedded for TEM processing, as described above. The fractured sample fragments were dehydrated through a graded series of ethanol washes, and critical point dried. The samples were subsequently sputter coated and imaged using a high-resolution field emission scanning electron microscope (Hitachi S-4800).

### ATP bioluminescence assay

FAMS samples for ATP generation assays comprised of size-gated total MTG-positive events (*n* = 3), or size-gated low-Δψ_m_ (FITC^+^PE^–^) and high-Δψ_m_ (FITC^+^PE^+^) subpopulations (*n* = 3), were collected, pelleted, and resuspended in prechilled mitochondrial respiration buffer^34^ (225 mM d-mannitol, 75 mM sucrose, 10 mM KCl, 10 mM Tris-HCl, 5 mM KH_2_PO_4_, pH 7.2). ATP standards and luciferase reagents were prepared from a standard ATP bioluminescence assay kit (Roche). To uncouple oxidative phosphorylation, samples were incubated in respiration buffer containing 10 µM FCCP (R&D Systems) for 5 min at room temperature, prior to analysis. To study the ability of isolated mitochondria to generate ATP, samples were incubated in respiration buffer containing 400 µM ADP (Cell Technology) and incubated for 10 min at room temperature prior to addition of the luciferase reagent. Luminescence was immediately read upon addition of luciferase using a Biotek Synergy H1 plate reader, and net ATP generation over assay controls was quantitated using a standard curve of serially diluted ATP. All biological replicates were run with technical duplicates of 5 × 10^5^ mitochondria events per well and statistical significance was determined by ANOVA with post-hoc Tukey HSD test and analyzed using Microsoft Excel (version 15.27) or Graphpad Prism (Version 7.0d)

### PCR evaluation of nuclear and mtDNA

Size gated, MTG-positive events (2.0 × 10^6^) sorted from mouse liver tissue was collected as described previously in three biological replicates. Sorted events were pelleted at 12,000 × *g* for 5 min at 4 °C and pellets were lysed in 50 μL mitochondrial lysis buffer (10 mM EDTA, 0.5% SDS, 0.1 mg mL^−1^ Proteinase K) at 45 °C for 1 h. Samples were diluted in nuclease-free water to limit amplification inhibition by the lysis buffer components. The presence of mitochondrial genes *mt-Nd1*, *mt-Nd2*, *mt-Nd5*, and *mt-Nd6*, and the nuclear gene, *Tert*, were assessed by PCR, using GoTaq Green (Promega; Supplementary Table 1).

### Mass spectrometry-based quantitative proteomics

Mitochondria were identified by size-gating and MTG, and sorted by FAMS as described above. For each tissue sample (liver and brain), three biological replicates of isolated mitochondria (~2.0 × 10^7^ events from liver, and 5.0 × 10^7^ events from brain per replicate) were collected and pooled from tissue obtained from four animals per replicate. The collected mitochondria were pelleted by repeated centrifugation and stored frozen at −80 °C until processing. The samples were lysed in a buffer containing 50 mM triethylammonium bicarbonate (TEAB), pH 8.0 with 1% SDS in mass spectrometry-grade water, vortexed, and sonicated in an ice-cold sonication bath for 10 min. Following sonication, the samples were centrifuged at 16,000 × *g* for 10 min at 4 °C, and the protein concentration of the supernatant was quantitated using 100× diluted sample aliquots and a micro-BCA assay (Pierce). Total protein for each sample was reduced with 10 mM TCEP for 30 min at room temperature and alkylated with 20 mM iodoacetamide for 30 min in the dark, followed by precipitation with cold acetone. The cleaned-up protein fraction (4.9 µg of total protein for each sample) was reconstituted in 25 mM TEAB, pH 8.0, and enzymatic digestion was conducted with sequencing-grade Lys-C (Wako) for 4 h at an enzyme/substrate (E:S) ratio of 1:50 and trypsin (Promega) at an E:S ratio of 1:50 with overnight digestion in a shaker at 37 °C. The resultant digests of mitochondrial isolates from liver and brain tissues were labeled with six stable isotope labeled tandem mass tags (six-plex TMT, Thermo Fisher Scientific), using the scaled down StageTip-based^36^ experimental protocol with a single stage elution^37^. The resultant TMT-labeled protein digests were combined and analyzed using a 5 h linear gradient a reversed-phase nanoflow liquid chromatography (RP nLC) column (75 µm × 25 cm) coupled with a Q Exactive tandem mass spectrometer (Thermo Fisher Scientific) in data-dependent acquisition mode. The raw MS data files from each nLC tandem mass spectrometry (nLC-MS/MS) run were processed using Proteome Discoverer (v. 2.1) (Thermo Fisher Scientific) for protein identification with two search engines: Sequest HT and Mascot. A fasta database containing both reviewed and nonreviewed mouse protein sequences and isoforms was downloaded from UniProt on 07/29/2016 and concatenated with protein sequences of common contaminants (cRAP)^38^. Protein identifications were filtered down to FDR of 0.01 based on *q*-values. The proteins with peptide spectral matches identified for reporter ions of all six TMT channels were considered as quantifiable proteins. Relative quantitation of proteins was achieved by the comparison of TMT reporter ion intensities among samples using the Proteome Discoverer software. Protein abundance values were normalized for each TMT channel by the sum of all intensity values measured for this particular channel. After the protein abundance ratios were determined, the average of each protein ratio (liver:brain) from the three separate biological replicates was used as a measure of differences between the two mitochondrial isolates. The ratio- and *p*-value-based filtering technique was applied to select the differentially expressed proteins with high confidence (absolute value of protein liver:brain abundance ratio >1.5, *p* < 0.05). The determined lists of differentially expressed proteins were subjected to a biological process overrepresentation test using a Fisher’s Exact test with false discovery rate correction in comparison to the *Mus musculus* reference database (Panther v14.0).

### Quantitative PCR for mtDNA copy number analysis

For analysis of mtDNA copy number in pooled mouse liver mitochondrial samples based on Δψ_m_ and size differences, 1 × 10^6^ mitochondria were collected and prepared for quantitative PCR (qPCR). Relative levels of *mt-Nd1* and *mt-Nd4* were assessed using TaqMan^®^ gene expression assays (Thermo Fisher; Supplementary Table 1), due the absence of the typical genomic DNA to use as a reference gene for the normalization of mtDNA copy number (Fig. 3c), we implemented a spike in control of λ phage DNA (Supplementary Table 1) to ensure equivalent amplification efficiency. Samples were added to TaqMan^®^ Fast Advanced MasterMix (Applied Biosystems), and run on a StepOnePlus Real-Time PCR system (Applied Biosystems) under standard fast reaction parameters. Fold change in gene levels was calculated relative to the total mitochondria population for *n* = 3 replicate experiments. Data were analyzed for significance by ANOVA followed by a post-hoc Tukey HSD test.

### Assessment of mtDNA copy number in individual mitochondria

Due to the sensitivity of smPCR for the amplification of low copy number templates, including potential contaminants (an initial pilot study revealed 4 out of blank 15 wells that contained an amplicon), our copy number analysis was performed under rigorous parameters, including a strategy in which mitochondria from two mouse strains (C57BL/6 and CD-1) were independently tagged with different fluorescent antibodies (C57BL/6: DyLight 650 and CD-1: AlexaFluor 568, as described above) and mixed prior to sorting (Fig. 6c). We then validated this strategy by matching the fluorescent label with a strain-specific C57BL/6 polymorphism, C9461T, via sequencing. Using this strategy, we confirmed 100% fidelity between the strain identity of fluorophore and polymorphism (Fig. 6d). We then applied this strategy to the genetic analysis of single mitochondria within differing size ranges. The total mitochondria population, identified by MTG, was bisected such that half of mitochondria events were gated as small (~0.2–0.5 µm) and half gated as large (~0.5–1 µm), as determined by size calibration particles (Fig. 6f), and samples were collected into individual wells of a 96-well PCR plate containing 1 µl of mitochondrial lysis buffer (10 mM EDTA, 0.5% SDS), immediately overlaid with mineral oil, and incubated at 37 °C for 1 h. Following lysis, plates were stored at −80 °C. For strain detection, primers (Supplementary Table 1) were designed around a C57BL/6-specific polymorphism, C9461T, to directly identify C57BL/6 mtDNA molecules. Lysed samples were diluted across 32 PCR wells to both prevent inhibitory effects of mitochondrial lysis buffer on amplification and to ensure templates were individually segregated into separate wells. Single molecule PCR (smPCR)^39,40^ was performed using Ex Taq DNA Polymerase, Hot Start Version (Takara) with LA PCR buffer (Takara) to generate 331 base pair amplicons. PCR products were individually sequenced using a 3720xl DNA Analyzer (Applied Biosystems) and analyzed with CodonCode Aligner software (CodonCode Corporation) to determine the strain identity of each mitochondrion based on strain-specific polymorphisms. Of note, the molecules sequenced from positive control mitochondria from the CD-1 mouse contained a G9348A polymorphism when initially screened for the presence of the C57BL/6-specific polymorphism. The presence of this polymorphism allowed us to further distinguish samples used in our analysis from any contaminating wild-type CD-1 mtDNA molecules (which contain a G10105A polymorphism). Sequences obtained from contaminating wild-type CD-1 mtDNA molecules were excluded from reported results. These exclusion criteria were not preestablished. Of the 162 mitochondria tested, 41 yielded no amplified product, 121 yielded at least one mtDNA molecule, of which 85 were sequenced to confirm strain identity. Investigators were blinded to group allocation of individual mitochondria during data collection for single molecule PCR, but blinding was not relevant to data collection or analysis for additional functional endpoints. The mtDNA copy number for small size-gated mitochondria was non-normally and nonidentically distributed according to the D’Agostino–Pearson omnibus K2 test for normality (*P* = 0.0037), as such, the nonparametric Mann–Whitney U one-tailed test was used to determine statistical significance between the small and large size-gated subpopulations in both strains. Analysis was done using Graphpad Prism (Version 7.0d, Graphpad Software, Inc.).

### Statistics and reproducibility

For most data sets significance was determined by ANOVA with post-hoc Tukey HSD test. For non-normally distributed data sets (Fig. 6g), as determined by the the D’Agostino–Pearson omnibus K2 test for normality, the nonparametric Mann–Whitney U one-tailed test was used to determine statistical significance. For most studies, a sample size of at least three biological replicates (as indicated in text) was used to determine statistical significance between test groups, however no sample-size calculation was performed. Using the described methodology, all attempts at replication were successful.

### Reporting summary

Further information on research design is available in the Nature Research Reporting Summary linked to this article.

## Supplementary information

Supplemental Information

Description of Supplementary Data

Supplemental Data 1

Reporting Summary

## Data Availability

The datasets generated from this study are included in this published article as Supplementary Data. All sequencing data generated from this study were deposited in the NCBI Sequence Read Archive with the project identifier PRJNA542698. The proteomics mass spectrometry data were deposited to the MassIVE repository, with the dataset identifier MSV000083837. The spectral data can be downloaded from the URL https://massive.ucsd.edu/ProteoSAFe/dataset.jsp?task=842821d25de94077aa3487b21106e394
