# Supplementary material for: A nanoscale, multi-parametric flow cytometry-based platform to study mitochondrial heterogeneity and mitochondrial DNA dynamics
Source: Commun Biol. 2019 Jul 11;2:258. doi: 10.1038/s42003-019-0513-4 (PMC6624292; doi:10.1038/s42003-019-0513-4)
Supplement: Supplementary file 1 — Supplemental Information [file 42003_2019_513_MOESM1_ESM.pdf]

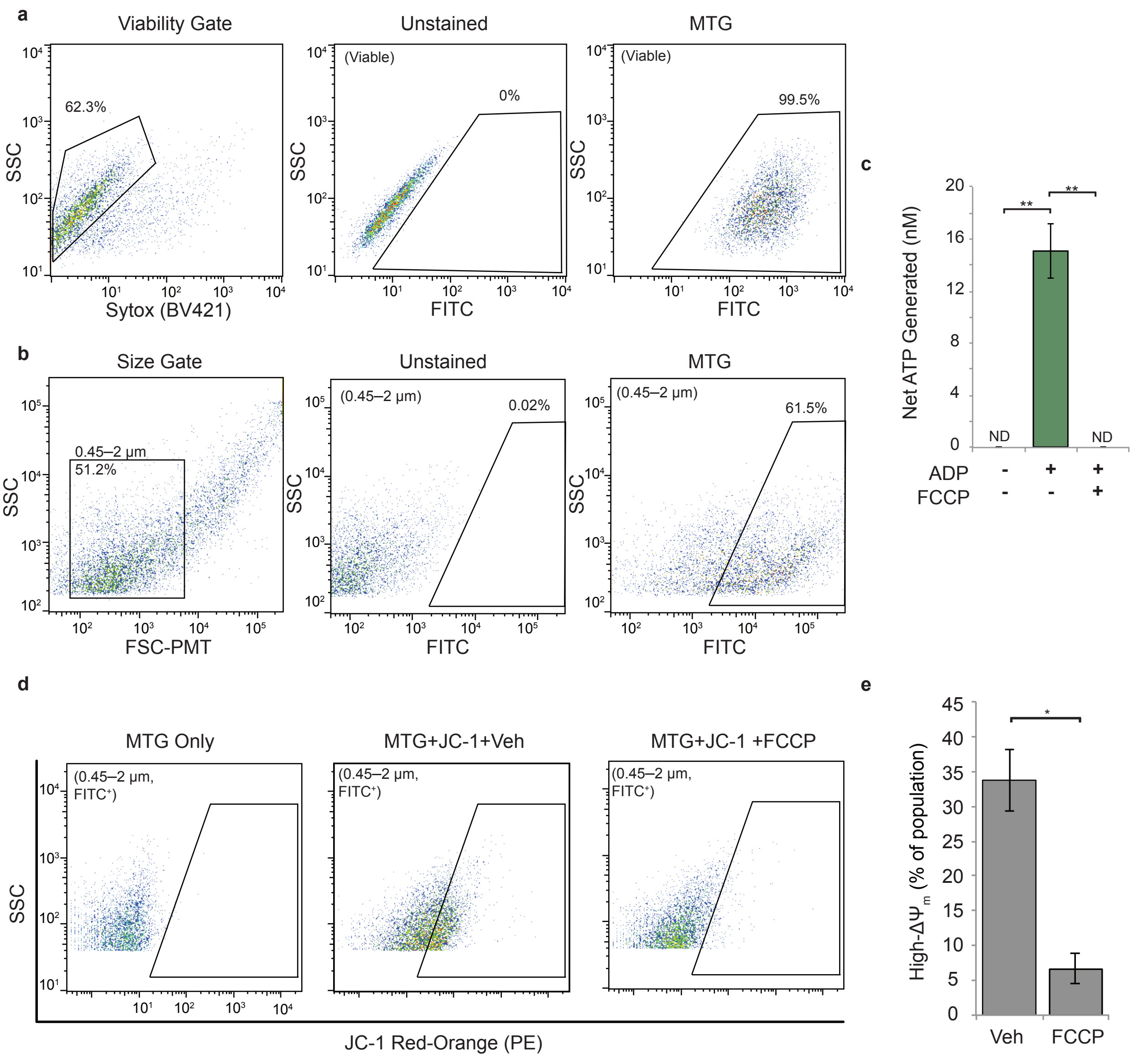

Supplementary Figure 1. Isolation of mitochondria from cultured mammalian cell lines using FAMS. (a) Murine embryonic fibroblasts (MEF) were labeled with MTG and analyzed by conventional FACS using Sytox Blue Dead Cell Stain to determine viability. (b) MEF were subsequently lysed and analyzed by FAMS for MTG-positive events within a 0.45-µm–2.0-µm size gate. (c) Size gated, MTG-positive events generated ATP in the presence of ADP while the addition of uncoupler, FCCP, decreased the signal below background. ND indicates ATP was not detected, mean ± SEM; n = 3 (P < 0.01**). (d) Cells of the human liver hepatocellular carcinoma cell line, HepG2, were labeled with MTG and JC-1. Size gated, FITC+ events were analyzed for JC-1 red-orange fluorescence to assess mitochondria with high-ΔΨm. The percent of FITC+PE+ (high-ΔΨm) was analyzed after treatment with vehicle (ethanol) or FCCP. (e) Average percent of high-ΔΨm mitochondria (n = 3; mean ± SEM shown; P < 0.05*).

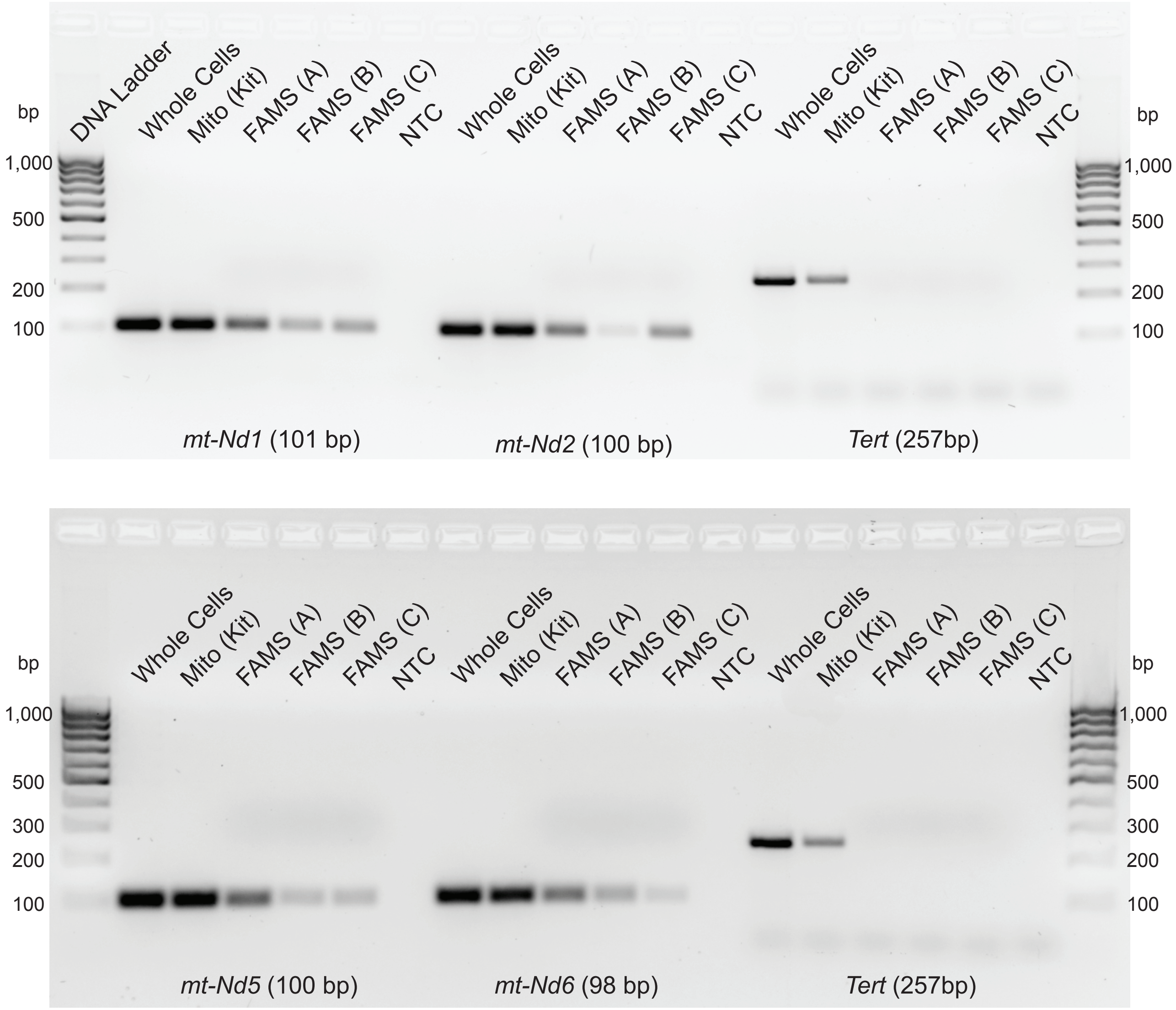

Supplementary Figure 2. Uncropped gel images for PCR products presented in Figure 3c. Size-gated, MTG-positive events (FAMS replicates A-C) expressed the mtDNA encoded genes, mt-*Nd1*, mt-*Nd2*, mt-*Nd5*, and mt-*Nd6*, but not *Tert*, a nuclear-encoded gene. Mitochondria isolated using a commercially available isolation kit (differential centrifugation) exhibit variable mtDNA purity. NTC, 'no template' control.

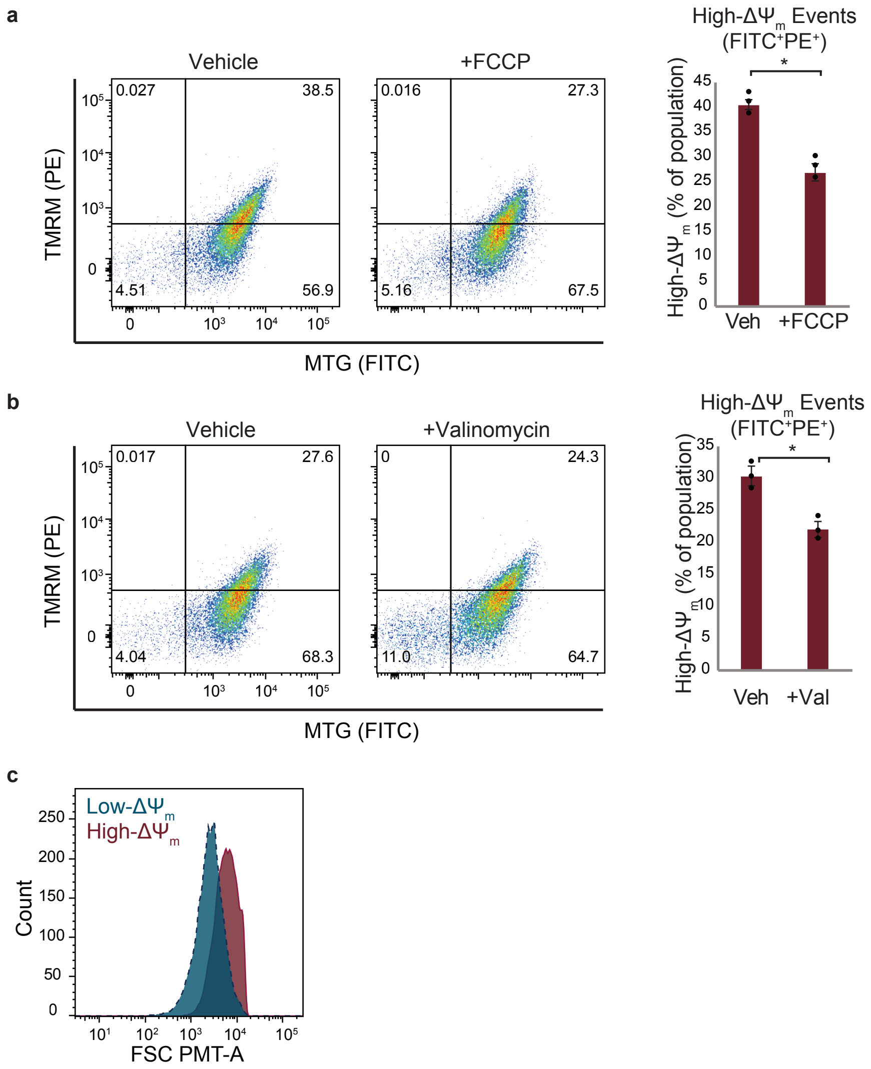

Supplementary Figure 3. Identification of mitochondrial subpopulations by ΔΨm, using TMRM. (a) After gating based on size and FITC+ [MTG] events, mitochondria with high-ΔΨm were identified as PE+. FCCP significantly reduced the number of high-ΔΨm mitochondria, mean ± SEM; n = 3 ($P < 0.05$*). (b) After gating based on size and FITC+ [MTG] events, mitochondria with high-ΔΨm were identified as PE+. Valinomycin significantly reduced the number of high-ΔΨm mitochondria, mean ± SEM; n = 3 ($P < 0.05$*). (c) High-ΔΨm and low-ΔΨm mitochondrial subpopulations were assessed for size distribution based on FSC-PMT (representative histogram for n = 3).

Supplementary Table 1. Primers used for PCR analyses.

Conventional PCR

Gene	Accession Number	Primer Sequence	
NADH dehydrogenase subunit 1 (mt-ND1)	NC_005089 REGION: 2751..3707	Forward	CAATTTACCAGAACTCTACTCAACTAAC
		Reverse	CGTAACGGAAGCGTGGATAA
NADH dehydrogenase subunit 2 (mt-ND2)	NC_005089 REGION: 3914..4951	Forward	CTATCACCCTTGCCATCATCTAC
		Reverse	CTGAATTCCAGGCCTACTCATATT
NADH dehydrogenase subunit 5 (mt-ND5)	NC_005089 REGION: 11742..13565	Forward	CTTATCCTCACCTCAGCCAAC
		Reverse	CGTCCGTACCATCATCCAATTA
NADH dehydrogenase subunit 6 (mt-ND6)	NC_005089 REGION: complement(13552..14070)	Forward	TGAGGTTGATGATGTTGGAGTT
		Reverse	CAAAGATCACCCAGCTACTACC
Telomerase reverse transcriptase (Tert)	NM_009354	Forward	TCTACCGCACTTTGGTTGCC
		Reverse	CAGCACGTTTCTCTCGTTGC

Single Molecule PCR

Template	Acccession Number	Primer Sequence	
Mus musculus mitochondrion	NC_005089.1	mtDNA:9203-9235	GGCTACTGGATTCCATGGACTCCATGTAATTAT
		mtDNA:9502-9534	GGGGGAGTCAGAATGCAACTAGAATTAGCGTTA
		mtDNA:10207-10235	GGTTTTTTTAGGGCTTGATAGTCAGGTTA

Quantitative PCR

Gene	Accesssion Number	Assay ID	
NADH dehydrogenase subunit 1 (mt-ND1)	NC_005089_ND1.0	Mm04225274_s1	
NADH dehydrogenase subunit 4 (mt-ND4)	NC_005089_ND4.0	Mm04225294_s1	
Control		Assay	
Lambda bacteriophage (cI857 Sam7)		Primer 1	CGCACAGGAACTGAAGAATG
		Primer 2	CCGTCGAGAATACTGGCAAT
		Probe	TGTACTTTCGTGCTGTCGCGGATCG
