# Supplementary material for: A nanoscale, multi-parametric flow cytometry-based platform to study mitochondrial heterogeneity and mitochondrial DNA dynamics
Source: Commun Biol. 2019 Jul 11;2:258. doi: 10.1038/s42003-019-0513-4 (PMC6624292; doi:10.1038/s42003-019-0513-4)
Supplement: Supplementary file 2 — Description of Supplementary Data [file 42003_2019_513_MOESM2_ESM.docx]

**Description of Additional Supplementary Files**

**File Name**: Supplementary Data 1

**Description**: An Excel file containing data source for all charts and graphs presented in the main figures, and the complete mass spectrometry proteomics data referenced in text.
