# Supplementary material for: A nanoscale, multi-parametric flow cytometry-based platform to study mitochondrial heterogeneity and mitochondrial DNA dynamics
Source: Commun Biol. 2019 Jul 11;2:258. doi: 10.1038/s42003-019-0513-4 (PMC6624292; doi:10.1038/s42003-019-0513-4)
Supplement: Supplementary file 4 — Reporting Summary [file 42003_2019_513_MOESM4_ESM.pdf]

nature research

Corresponding author(s): Woods, Dori

Reporting Summary

Nature Research wishes to improve the reproducibility of the work that we publish. This form provides structure for consistency and transparency in reporting. For further information on Nature Research policies, see Authors & Referees and the Editorial Policy Checklist.

Statistical parameters

When statistical analyses are reported, confirm that the following items are present in the relevant location (e.g. figure legend, table legend, main text, or Methods section).

n/a	Confirmed	
☐	☒	The exact sample size (*n*) for each experimental group/condition, given as a discrete number and unit of measurement
☒	☐	An indication of whether measurements were taken from distinct samples or whether the same sample was measured repeatedly
☐	☒	The statistical test(s) used AND whether they are one- or two-sided<br>*Only common tests should be described solely by name; describe more complex techniques in the Methods section.*
☒	☐	A description of all covariates tested
☐	☒	A description of any assumptions or corrections, such as tests of normality and adjustment for multiple comparisons
☐	☒	A full description of the statistics including central tendency (e.g. means) or other basic estimates (e.g. regression coefficient) AND variation (e.g. standard deviation) or associated estimates of uncertainty (e.g. confidence intervals)
☒	☐	For null hypothesis testing, the test statistic (e.g. *F*, *t*, *r*) with confidence intervals, effect sizes, degrees of freedom and *P* value noted<br>*Give P values as exact values whenever suitable.*
☒	☐	For Bayesian analysis, information on the choice of priors and Markov chain Monte Carlo settings
☒	☐	For hierarchical and complex designs, identification of the appropriate level for tests and full reporting of outcomes
☒	☐	Estimates of effect sizes (e.g. Cohen's *d*, Pearson's *r*), indicating how they were calculated
☐	☒	Clearly defined error bars<br>*State explicitly what error bars represent (e.g. SD, SE, CI)*

Our web collection on statistics for biologists may be useful.

Software and code

Policy information about availability of computer code

Data collection	Data were collected using the following commercially available software: BD FACSDiva software (version 8.0.1), Image Lab (version 5.0)
Data analysis	Data were analyzed using the following commercially available software:Microsoft Excel (version 15.27), Graphpad Prism (Version 7.0d), ImageJ (version 1.49), FlowJo (version 10).

For manuscripts utilizing custom algorithms or software that are central to the research but not yet described in published literature, software must be made available to editors/reviewers upon request. We strongly encourage code deposition in a community repository (e.g. GitHub). See the Nature Research guidelines for submitting code & software for further information.

Data

Policy information about availability of data

All manuscripts must include a data availability statement. This statement should provide the following information, where applicable:

- Accession codes, unique identifiers, or web links for publicly available datasets
- A list of figures that have associated raw data
- A description of any restrictions on data availability

The datasets generated from this study are included in this published article as Supplementary Data. All sequencing data generated from this study was deposited in the NCBI Sequence Read Archive with the project identifier PRJNA542698. The mass spectrometry proteomics data have been deposited to the MassIVE database

hosted by the Center for Computational Mass Spectrometry at University of California, San Diego (http://massive.ucsd.edu) with the dataset identifier MassIVE MSV000083837.

Field-specific reporting

Please select the best fit for your research. If you are not sure, read the appropriate sections before making your selection.

☒ Life sciences ☐ Behavioural & social sciences ☐ Ecological, evolutionary & environmental sciences

For a reference copy of the document with all sections, see nature.com/authors/policies/ReportingSummary-flat.pdf

Life sciences study design

All studies must disclose on these points even when the disclosure is negative.

Sample size	For most studies, a sample size of at least 3 biological replicates was used to determine statistical significance between test groups, however no sample-size calculation was performed.
Data exclusions	PCR products from single molecule PCR analyses were individually sequenced using a 3720xl DNA Analyzer (Applied Biosystems) and analyzed with CodonCode Aligner software (CodonCode Corporation) to determine strain identity of each mitochondrion based on strain-specific polymorphisms. Of note, the molecules sequenced from positive control mitochondria from the CD-1 mouse contained a G9348A polymorphism when initially screened for the presence of a C57BL/6-specific polymorphism (C9461T). The presence of this polymorphism allowed us to distinguish samples used in our analysis from any contaminating wild-type CD-1 mtDNA molecules (which contain a G10105A polymorphism). Sequences obtained from contaminating wild-type CD-1 mtDNA molecules were excluded from reported results. These exclusion criteria were not pre-established.
Replication	Using the described methodology, all attempts at replication were successful.
Randomization	For all endpoints, isolated mitochondria, were allocated into experimental groups through non-random sorting criteria, as described in text.
Blinding	Investigators were blinded to group allocation of individual mitochondria during data collection for single molecule PCR, but blinding was not relevant to data collection or analysis for additional functional endpoints.

Reporting for specific materials, systems and methods

Materials & experimental systems

n/a	Involved in the study	
☒	☐	Unique biological materials
☐	☒	Antibodies
☐	☒	Eukaryotic cell lines
☒	☐	Palaeontology
☐	☒	Animals and other organisms
☒	☐	Human research participants

Methods

n/a	Involved in the study	
☒	☐	ChIP-seq
☐	☒	Flow cytometry
☒	☐	MRI-based neuroimaging

Antibodies

Antibodies used	Rabbit polyclonal anti-TOM20 (Santa Cruz Biotechnology; sc-11415, clone FL-145, Lot#L1713)<br>Mouse monoclonal anti-TRAP1 (Abcam; ab2721, Lot#GR219935-6)<br>goat anti-mouse AlexaFluor 647 (CST Molecular Probes; 4410S, Lot#10)<br>goat anti-rabbit AlexaFluor 568 (Life Technologies; A11011, Lot#1942295)
Validation	Primary antibodies against TOM20 and TRAP1 were validated by the manufacturers using positive control Western blots (by size) in both human and mouse samples

Eukaryotic cell lines

Policy information about cell lines

Cell line source(s)	HepG2 was obtained from ATCC (ATCC® HB-8065™) and maintained as recommended by the supplier.<br>CF-1 primary mouse embryonic fibroblasts (MEF) were obtained from MTI-GlobalStem (GSC-6001) and maintained as recommended by the supplier.
Authentication	None of the cell lines used were authenticated.
Mycoplasma contamination	Cell lines were not tested for mycoplamsa contamination
Commonly misidentified lines (See ICLAC register)	Neither cell line is included in the ICLAC register for commonly misidentified lines

Animals and other organisms

Policy information about studies involving animals; ARRIVE guidelines recommended for reporting animal research

Laboratory animals	Female C57BL/6 or male CD-1 mice were obtained from Charles River Laboratories
Wild animals	Study did not involve wild animals
Field-collected samples	Study did not involve samples collected from the field

Flow Cytometry

Plots

Confirm that:

☒ The axis labels state the marker and fluorochrome used (e.g. CD4-FITC).

☒ The axis scales are clearly visible. Include numbers along axes only for bottom left plot of group (a 'group' is an analysis of identical markers).

☒ All plots are contour plots with outliers or pseudocolor plots.

☒ A numerical value for number of cells or percentage (with statistics) is provided.

Methodology

Sample preparation

Animals and cell lines
All animals utilized for this study were young adult (8-12-week-old) female C57BL/6 or male CD-1 mice (Charles River Laboratories), and all experiments described herein were reviewed and approved Institutional Animal Care and Use committee at Northeastern University. Mouse tissues were harvested and immediately dissociated into single-cell suspensions for mitochondrial preparations. The human liver hepatocellular carcinoma cell line, HepG2 was obtained from ATCC (ATCC® HB-8065™) and maintained as recommended by the supplier. CF-1 mouse embryonic fibroblasts (MEF) were obtained from MTI-GlobalStem (GSC-6001) and maintained as recommended by the supplier. All reagents were purchased from Thermo Fisher Scientific unless otherwise noted.

Tissue dissociation into single cell suspension
Harvested tissues (300–1,200 mg) were rinsed briefly in warm Hank's balanced salt solution with calcium and magnesium (HBSS +/+) and minced into small pieces. For MitoTracker™ Green FM (MTG; LifeTechnologies) labeling experiments, minced tissue (liver, heart, spleen, brain, or kidney) was placed in PBS containing 0.5% bovine serum albumin and 2 mM EDTA ("PEB Buffer") and mechanically dissociated using a gentleMACS Tissue Dissociator (Miltenyi). For further subpopulation analyses and sample collection for proteomics analyses, liver or brain tissue was minced and placed in a tissue-specific dissociation mixture. Liver dissociation buffer contained 500 U/ml collagenase IV (Worthington Biochemical Corporation) and 150 U/ml DNaseI (Sigma Aldrich) in HBSS+/+, and brain dissociation buffer contained PBS with 2 mM EDTA. Tissues were incubated at 37°C with continuous agitation for 30 minutes and then mechanically dissociated using a gentleMACS Tissue Dissociator. All samples were filtered through a 100 µm nylon mesh cell strainer, rinsed with PEB buffer, and maintained at 4°C prior to labeling.

MitoTracker™, DAPI, and JC-1 labeling
Cultured cells were trypsin digested and brought to a single cell suspension in PBS containing 1% bovine serum albumin and 2.5 mM EDTA (FACS Buffer). Whole cells were stained with 100 nM MTG, 2 µM JC-1 (Marker Gene Technologies), or both dyes simultaneously at 37°C for 15 minutes. Dispersed liver or brain tissue was stained with 100 nM MTG for sorting whole populations of mitochondria. After mitochondrial labeling, cell suspension samples were briefly pelleted and resuspended in ice-cold cell lysis buffer containing 300 mM sucrose, 10 mM Tris pH 7.4, 0.5 mM EDTA, and 1X Halt Protease Inhibitor Cocktail in PBS. Tissue samples were lysed by vortexing, while HepG2 and MEF cells were dounce homogenized on ice to complete lysis.
In other studies, a single cell suspension from mouse liver tissue was stained with 25 nM MitoTracker™ Red CMXRos (MTR; Life Technologies), and lysed as described above. Lysed cell samples were fixed in 2% paraformaldehyde (PFA) for 5 minutes at room temperature, rinsed with PBS, and subsequently stained with 3 µM 4',6-Diamidino-2-Phenylindole, Dihydrochloride (DAPI) for 5 minutes at room temperature. After a final rinse, samples were analyzed for staining of mtDNA within the population of size-gated MTR+ events.

For mitochondrial subpopulation sorting from liver tissue, dispersed cell suspensions were simultaneously stained with 25 nM MTG and 1 µM JC-1 for 15 minutes at 37°C, 5% CO_2. To test the specificity of JC-1 labeling, stained cells were incubated with 10 µM carbonyl cyanide 4-(trifluoromethoxy)phenylhydrazone (FCCP; Tocris) for 15 minutes at 37°C, 5% CO_2, prior to lysis.

Antibody labeling
A single cell suspension from mouse liver tissue was stained with 25 nM MTG as described above and lysed in cell lysis buffer. Samples were centrifuged at 12,000 x g for 5 minutes at 4°C, resuspended in blocking buffer (2% bovine serum albumin, 2% normal goat serum in PBS), and incubated on crushed ice for 20 minutes. Samples were centrifuged at 12,000 x g, and resuspended in anti-TOM20 (Santa Cruz Biotechnology; sc-11415, 1:10), anti-TRAP1 (Abcam; ab2721, 1:50), or both antibodies together. Samples were washed in ice cold PBS and resuspended in goat anti-mouse AlexaFluor 647 (Molecular Probes, 1:250), goat anti-rabbit AlexaFluor 568 (Life Technologies, 1:250), or both antibodies together and incubated on ice for 20 minutes. Wash steps were repeated and samples were resuspended in cold sheath fluid for FAMS analysis. In a separate experiment to identify mitochondria from a mixed pool of mouse strains, mouse liver homogenates from CD-1 and C57BL/6 strains incubated with TOM20 (1:50), followed by incubation with either AF568 (for CD-1) or DL650 (for C57BL/6) secondary antibodies. The samples were washed, and then combined at a 1:1 ratio, and resuspended in cold sheath fluid for FAMS analysis.

Instrument

All analysis was completed using a special-order research product BD FACS Aria III, fitted with a photomultiplier tube (PMT) detector for forward light scatter (FSC)

Software

BD FACSDiva software (version 8.0.1), FlowJo (version 10).

Cell population abundance

No cells were sorted in this study

Gating strategy

To achieve high-resolution detection of subcellular-sized calibration beads (< 1 µm), the instrument detection threshold was routinely set to side light scatter (SSC) 200. The use of fluorescently labeled size calibration particles (Spherotech) allowed for robust identification of subcellular-sized particles in what would otherwise be considered the debris field, or instrument noise, under standard cell sorting parameters (Fig. 1a,b). Following calibration of laser area scaling and delay, for each sort, a mixture of size calibration beads (Spherotech and Life Technologies) was run to optimize light scatter voltages to visualize events between 220 nm and 6 µm, with typical size gates for sorts of mouse liver mitochondria capturing particles ranging in size from 450 nm to 2 µm, based on TEM evaluation of mitochondria size in situ (Supplementary Figure 1). Mitochondria were identified by size, and positive fluorescence of mitochondria-specific dyes, such as MitoTracker™ (Life Technologies), or secondary antibody fluorophore, with respect to unstained negative control samples or control samples labeled with the secondary antibody alone.

☒ Tick this box to confirm that a figure exemplifying the gating strategy is provided in the Supplementary Information.
